# Factors influencing first childbearing timing decisions among men: Path analysis

**Published:** 2016-09

**Authors:** Nourossadat Kariman, Maliheh Amerian, Padideh Jannati, Fatemeh Salmani

**Affiliations:** 1 *Department of Midwifery and Reproductive Health, Nursing and Midwifery Faculty, Shahid Beheshti University of Medical Sciences, Tehran, Iran.*; 2 *Department of Midwifery, School of Nursing and Midwifery, Shahrood University of Medical Sciences, Shahrood, Iran.*; 3 *Department of Statistics, Paramedical Sciences Faculty, Shahid Beheshti University of Medical Sciences, Tehran, Iran.*

**Keywords:** *Childbearing*, *Decision-making*, *Influencing factors*, *Paternal*, *Men*

## Abstract

**Background:**

Factors that influence men’s childbearing intentions have been relatively unexplored in the literature.

**Objective:**

This study aimed to determine the influencing factors about the first childbearing timing decisions of men.

**Materials and Methods:**

In this cross-sectional study, 300 men who were referred to private and governmental healthcare centers in Shahrood, Iran were randomly recruited from April to September 2014. Data were collected using a demographic questionnaire, the Quality of Life Questionnaire; ENRICH Marital Satisfaction Questionnaire, Synder’s Hope Scale, and the Multidimensional Scale of Perceived Social Support.

**Results:**

After removing the statistically insignificant paths, men’s age at marriage had the highest direct effect (β=0.86) on their first childbearing decision. Marital satisfaction (β=-0.09), social support (β=0.06), economic status (β=0.06), and quality of life (β=-0.08) were other effective factors on men’s first childbearing decisions. Moreover, marital satisfaction and social support had significant indirect effects on men’s childbearing decisions (β=-0.04 and -0.01, respectively).

**Conclusion:**

Many factors, including personal factors (age at marriage and quality of life), family factors (marital satisfaction), and social factors (social support), can affect men’s decision to have a child. Policymakers are hence required to develop strategies to promote the socioeconomic and family conditions of the couples and to encourage them to have as many children as they desire at an appropriate time.

## Introduction

With the increasing tendency to postpone childbearing, delayed childbearing has turned into a key characteristic of recent fertility trends in developed countries (1). Meanwhile, a steady increase in the number of children born to 30-49-year-old fathers and a constant reduction in the number of those born to 25-29-year-old men (from 123.1 per 1000 men in 1980 to 104.7 per 1000 men in 2005) has been reported in developed countries since 1980 (2, 3). In England and Wales, the birth rate in men older than 35 years increased from 25% of live births in 1993 to 40% in 2003. This rate has increased by 40% in the USA since 1980 (4). Moreover, in 24 out of 31 provinces of Iran, the total fertility rate has dropped to a value below the fertility replacement level (1, 2). Couples may decide to postpone childbearing due to a variety of reasons. Miles *et al* introduced effective birth control, higher numbers of educated and working women, value and partnership changes, improvement in gender equity, inappropriate housing and economic conditions, and the absence of family support programs as the main causes of delayed childbearing (6).

While numerous studies have evaluated the effects of advanced maternal age on obstetric and infant outcomes, the role of fathers has been generally neglected (7). However, growing evidence has suggested relationships between advanced paternal age and changes in the production of semen and sex hormones, sexual function, and fertility (3, 8). Advanced paternal age may also lead to subfertility, adverse pregnancy outcomes), birth defects, and nervous system cancers in adulthood (3). Possible mechanisms for these problems include single-gene mutations, autosomal dominant diseases, structural abnormalities in sperm chromosomes (e.g., reciprocal translocations) and multiple genetic/chromosomal defects (8, 9). Male factors other than age, e.g. occupation, environmental exposures to harmful substances, and smoking, have also been shown to affect sperm quality and cause early embryo loss (9).

Since advanced parental age is believed to affect pregnancy outcomes, the determinants of the age at which couples decide to have children need to be clarified. According to previous researches, men’s intentions and desires play a major role in determining the time of the first pregnancy and women’s decision to have a child (10, 11). Studies of couples’ intentions of childbearing have mainly focused on young men and women, especially students. The vast majority of women and men who attend university in Sweden, Finland, England, Canada, USA, and Australia wish to have children, preferably two, and they most commonly intend to have their first baby in their late 20s and early 30s (12-16). Some individuals, mainly men, even plan to have their first child at the age of 35 or later (17).

While men are known to exert both direct and indirect effects on women’s reproductive health, such effects have not been clearly discussed. The 1994 International Conference on Population and Development (ICPD) program of action indicated that increasing men’s contribution to women’s reproductive health needs effective education to promote their use of contraceptives, their support of women’s sexual and reproductive decisions, particularly birth control, and their responsible sexual behaviors to prevent and control sexually transmitted diseases (STDs) (18). 

it has been shown that many young men and women have limited knowledge about fertility problems at older ages and tend to be unrealistically optimist about the effectiveness of assisted reproductive technologies such as in vitro fertilization (IVF) (12, 15, 19, 20). Nevertheless, rather than being solely attributable to poor knowledge and optimistic biases among young adults, postponed parenthood is affected by a myriad of sociocultural and environmental factors (1). Therefore, in order to complement the existing epidemiological research, it is necessary to examine decision-making related to the age of childbearing from the perspectives of both men and women. 

The present study was hence conducted to fill the existing research gap by evaluating men’s reproductive preferences and intentions.

## Materials and methods

In this cross-sectional study, 360 eligible men (age: 18-45 years) were randomly selected. The subjects were married Iranian men who presented to healthcare facilities and private clinics in Shahrood, Iran along with their wives. The participants were selected using multi-stage cluster sampling during April to September 2014. The study protocols were approved by the Ethics Committee of Shahid Beheshti University of Medical Sciences, Tehran, Iran (No. 1393-1-86-13214). Before visiting the healthcare centers in Shahrood, The participants were provided with details about research objectives, ensured about data confidentiality, and asked to sign an informed consent form. Finally, participants filled out the questionnaires individually in a quiet room where the study was carried out.

The participants were excluded from the study if the couple had a history of infertility or if they provided incomplete questionnaires. Initially, healthcare centers in the urban area of Shahrood were divided into two categories, i.e. northern and southern centers. In each category, the urban centers were considered as clusters. Afterward, some healthcare centers were selected using simple random sampling and the sample size within each center was calculated using quota sampling based on the population they covered. Finally, the subjects were randomly selected from each center. The sampling was random, continuous and gradual in private clinics located in city center. The required sample size was calculated as 360 men using Cochran’s formulas with 0.2 error and 20% dropout rate (21).

Of the 360 questionnaires, 60 incomplete forms were removed and 300 completely filled questionnaires were considered as the final sample. The data were collected using the Quality of Life Questionnaire, adopted from the World Health Organization Quality of Life (WHOQOL), ENRICH (evaluation and nurturing relationship issues, communication, and happiness) Marital Satisfaction Scale, Snyder’s Hope Scale, the Multidimensional Scale of Perceived Social Support, and a demographic questionnaire. The demographic questionnaire contained items on the participants and their spouses’ age and education level, the subjects’ place of birth, place of residence, age at marriage, and duration of marriage, and their ideas about the perfect birth spacing and desired number of children.

The face and content validity of the brief WHOQOL has been previously assessed. The reliability of the questionnaire was also confirmed by calculating Cronbach’s alpha between 0.55 and 0.84 (22). The validity and reliability of ENRICH Marital Satisfaction Scale were also measured using content validity and Cronbach’s alpha (α=0.86) (23). The validity of Snyder’s Hope Scale was reported to be appropriate by using content validity (24). Snyder *et al* and Ghobari *et al* evaluated the reliability of the scale and reported Cronbach alpha coefficients equal to 0.81 and 0.88, respectively (25). The content validity of the Multidimensional Scale of Perceived Social Support was confirmed using principal components analysis. The reliability of the scale (Cronbach’s alpha) was also reported as 0.86-0.9 for the subscales and 0.86 for the whole scale (26). The reliability of the present research tool was assessed using test-retest and internal consistency. The correlation coefficients between the scores of Quality of Life Questionnaire, ENRICH Marital Satisfaction Scale, Snyder’s Hope Scale, and the Multidimensional Perceived Social Support were 0.73, 0.85, 0.89, and 0.76, respectively. The corresponding Cronbach’s alpha coefficients were 0.84, 0.76, 0.78, and 0.82, respectively.

**Statistical analysis**

The data were analyzed using SPSS 19 (SPSS Inc., Chicago, IL, USA) and IBM SPSS AMOS V 20 (USA). Descriptive statistics were applied to present and describe the data, create tables, and calculate the percentage, mean, and standard deviation values. Inferential statistics were used for analyzing the data and examining the relationships. Correlations between the variables were studied using Pearson’s correlation coefficients. Regression models were employed to examine relationships between dependent and independent variables. The direct and indirect effects of variables on age at the first childbearing decisions of men were evaluated using path analysis (p˂0.05).

## Results

The participants’ age, age at marriage, and duration of marriage were 28.8±3.74, 25.59±3.68, and 3.17±1.68 years, respectively. More than 40% of participants were university graduated and 45.3% were employees. Most subjects (92.3%) lived in urban areas and 43% of them were tenants. The monthly income of 38% of participants was 7-10 million Rials. Only 15% of the studied men reported a monthly income of over 10 million Rials. The mean number of desired children was 2.56±1.10 and the contraceptive method of choice was condoms in 47.0% of the participants. Table I shows the mean and standard deviation of key research variables.

Table II presents the correlation matrix of key variables to help specify the path analysis model. As seen, there were significant positive correlations (p<0.001) between age at the first childbearing decision and the participants’ age at marriage (r=0.89), duration of marriage (r=0.29), education (r=0.16), perceived social support (r=0.11), and economic status (r=0.27). However, age at the first childbearing decision was not significantly correlated with hope, desired number of children, and desired birth spacing. Meanwhile, there were significant negative correlations (p<0.001) between age at the first childbearing decision and other variables like marital satisfaction (r=-0.256) and quality of life (r=-0.220). 

The present study employed path analysis because the research objective was to investigate the mediatory and predictive role of variables (i.e. their direct, indirect, and total effects) and variance explained by the variables. Several indices were used to examine the model. The root mean square error of approximation (RMSEA) was less than 0.08 and comparative fit index (CFI), normed fit index (NFI), goodness of fit index (GFI), and adjusted goodness of fit index (AGFI) were all above 0.90. Therefore, the model was well-fitting. The value of relative chi-square (chi-square divided by degree of freedom), i.e. 1.64 (<3), was acceptable and indicated a good-fitting model (Table III). 

**Figure 1:**
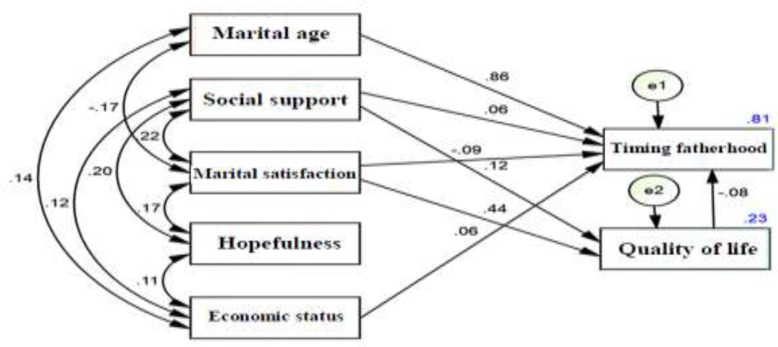
The results of experimental model of path analysis of factors associated with first childbearing decision. * Numbers on paths: path coefficient or factor beta weights (all pathcoeficients were significant at the 0.05 level). * Numbers on rectangles: explained variance

Figure 1 depicts the structured fitted model of the study population resulted from path analysis based on regression analysis. All paths except hope were significant and highlighted the effects of personal factors (age at marriage and quality of life), family factors (marital satisfaction), and social factors (social support) on first childbearing decision of men. Table IV summarizes the direct, indirect and total effect of each variable on the mediator and dependent variables. The results showed that marital age, marital satisfaction, social support, economic status, and quality of life had direct effects on men’s age at the first childbearing decision. Age at marriage had the greatest effect and played the most important role. Moreover, marital satisfaction and social support had both direct and indirect effects on age at the first childbearing decision. The results showed that hope did not have a direct effect on men’s age at the first childbearing decision. Hence, considering its insignificance, this path was removed from the diagram in Figure 1.

**Table I:** Distribution of factors scores in men who referred to private and governmental health clinics, Shahrood City, 2014

**Variable **	**Mean**	**Standard deviation**	**Maximum**	**Minimum**
Paternal age(year)	28.80	3.78	42	18
Age at marriage(year)	25.59	3.68	40	14
Marital satisfaction(175)*	118.43	26.74	170	49
Hope(32)*	19.54	4.82	32	11
Perceived social support(84)*	58.77	13.10	84	14
Quality of life(130)*	96.64	12.31	125	35

* Maximum score of questionnaire

**Table II:** Correlations between men’s age at the first childbearing decision and marital satisfaction, social support, hope, quality of life, and economic status

**Variable**	**Age at the first childbearing decision**	**Marital satisfaction**	**Social support**	**Hope**	**Quality of life**	**Economic status**
Age at the first childbearing decision	1					
Marital satisfaction	-0.256*	1				
Social support	0.118*	0.197	1			
Hope	-0.016*	0.161*	0.020*	1		
Quality of life	-0.220*	0.462*	0.298*	0.182*	1	
Economic status	0.217*	-0.094	-0.103	0.100	-0.006	1

* All correlation coefficients are significant at the 0.001 level.

**Table III:** Goodness of fit indices of factors associated with the first childbearing decision

**Model index**	**χ** ^2^	**df**	**RMSEA**	**GFI**	**NFI**	**CFI**
	11.47	7	0.046	0.99	0.98	0.99

**Table IV:** Factors associated with the first childbearing decision

**Variables**	**Effect**			**Path coefficient**	**P- value**
	**Direct**	**Indirect**	**Total**		
Age at marriage	0.86	-	0.86	0.856	0.000
Marital satisfaction	-0.09	-0.04	-0.13	-0.059	0.004
Social support	0.06	-0.01	0.05	0.057	0.028
Economic status	0.07	-	0.07	0.171	0.012
Quality of life	-0.08	-	-0.08	-0.069	0.006

* E_1_, E_2_: error variable.

## Discussion

The results of regression-based path analysis showed that men’s education had a direct impact on their decisions about the age of childbearing. Likewise, Adibi Sede *et al* indicated that men’s level of education had a negative impact on fertility rates (27). Kreyenfeld and Anderson assessed the data obtained from the German and Danish socioeconomic panel and reported the rate of childbearing to be significantly lower in unemployed people with higher education than in people with lower education (28). 

The present study found a significant direct correlation between men’s first childbearing decision and their age at marriage and duration of marriage. In fact, marriage at a younger age was associated with an earlier decision to have the first child. Among the various factors, age at the first marriage has been confirmed as an important confounding factor and the main determinant of the intentions to have the first child (7, 28). 

The present study showed a significant direct correlation between men’s age at the time of decision to have a child and their economic status. In other words, more desirable economic status of the family has a positive effect on childbearing decision-making in men. Razeghi-Nasrabad introduced the spouse’s employment and job as two important determinants of the interval between marriage and decision to have a child (29). Hezarjaribi and Abbaspour reported economic status to have a negative effect on women’s fertility (30). In other words, family’s better economic status reduced women’s fertility. 

Kreyenfeld and Anderson found a very strong relationship between men’s unemployment and the first childbearing decision. However, women’s unemployment was not related with their first childbearing intentions (28). In a study by Adair, increments in age and income had strong direct relationships with higher in interest and motivation for childbearing (31). Nilsen *et al* revealed a negative correlation between low socioeconomic status and age of first childbirth (7). Several studies have also highlighted financial insecurity as a major reason for delayed childbearing (7, 9, 16, 17). In a study by Thompson and Lee, Australian men reported financial security and a permanent stable job as main prerequisites for parenthood (1). 

According to our findings, marital satisfaction decreased the age at first childbirth. Several studies have similarly indicated a strong relationship between people’s perception of the quality of their relationships and their childbearing decisions with the intention to strengthen their married life and make efforts to prepare themselves (32). In fact, some couples consider childbirth in early years of their marriage as a great threat for their time to be together. Their fear of losing their freedom, leisure time, and travelling opportunities would thus result in their delayed childbearing. Another explanation for delayed childbearing is the lack of a comprehensive understanding of the spouse (which particularly happens in traditional marriages) and a sense of doubt about the stability of the marriage, especially when couples have frequent arguments during the first years of their marriage (5, 33, 34). 

The current study did not show a significant relationship between hope and men’s age at the first childbearing decision. In contrast, Nilsen *et al* investigated the effects of personal and social factors on childbearing decisions among Norwegian men at older ages and found a significant positive correlation between history of depressive symptoms and men’s age at the first childbirth (7). The results from another experimental study suggested the motivation for and interest in parenthood as a strong predictor for childbearing decisions. This inconsistency might be jsutified by different in sociocultural conditions of the studied societies (1, 3, 35). 

We also found quality of life is an important determinant of men’s decisions to have a child. Thompson and Lee reported preparedness, personal growth and maturity, financial security, and having a permanent stable job as prerequisites of parenthood among young Australian men (1). Likewise, Benzies *et al* underscored preparedness, physical health, and absence of chronic diseases as important factors in childbearing decisions (36). Several studies have identified perceived physical and emotional preparedness (i.e. feeling that an ideal environment for having and raising a child has been prepared), the ability to accept parental responsibilities, and awareness of risks associated with delayed fatherhood as important factors in childbearing decisions (1, 7, 17, 35).

There was a significant direct relationship between men’s social support and age at the first childbearing decision in the present study. In fact, men who enjoyed greater social support were more likely to postpone childbearing. The increasing number of studies on social support, highlighting its positive impacts and the negative consequences of its absence, show the significance of social factors in childbearing (5, 37, 38). Kalantari *et al* concluded that social participation decreased young people’s tendency toward childbearing (39). In Germany, Keim *et al* showed the influence of family members, friends, and relatives on couples’ childbearing decisions (40). Benzies suggested that the preparedness of the partner and the family played a less significant role in childbearing decisions (36). Contrary to our findings, Mill failed to establish a relation between delayed childbearing and a lack of support from family and relatives. This inconsistency might have been caused by differences in the sociocultural conditions of the two societies, differences in data collection tools, and different age groups of the participants (41).

Based on our findings, socioeconomic and family changes and the consequent changes in values, attitudes, motivations, and beliefs would affect not only people’s marriage and childbearing, but also their personal behaviors and beliefs. Generally, theories aiming to increase fertility cannot be implemented without the provision of favorable socioeconomic and psychological conditions in the society. The variables included in the regression analysis of factors associated with childbearing decisions explained about 80% of the total variance in fertility. 

Although this determination coefficient shows the high accuracy of the selected variables, these findings are limited to Shahrood and there is no strong evidence that such relationships might exist across the country. The novelty of the present study rests in providing a pathway analysis model for factors influencing childbearing timing decisions in men for the first time.

## Conclusion

This study confirmed the relationship between age at childbearing and factors such as hope, marital satisfaction, quality of life, age at marriage, social support, and economic status. Age at marriage played the most significant role in this regard. Social support and marital satisfaction had both direct and indirect (via quality of life) effects on age at the first childbearing decision. Considering the discussed barriers and problems to childbearing, policymakers need to encourage couples to have children by developing strategies to promote their family and socioeconomic conditions. Such strategies would reduce the effects of delayed childbearing and prevent further decline in fertility rates.

## Note

***This article extracted from M.Sc. thesis. (Maliheh Amerian)***
